# Effectiveness of adjuvant trastuzumab in daily clinical practice

**DOI:** 10.2478/raon-2013-0081

**Published:** 2014-11-05

**Authors:** Erika Matos, Branko Zakotnik, Cvetka Grasic Kuhar

**Affiliations:** Institute of Oncology Ljubljana, Department of Medical Oncology, Ljubljana, Slovenia

**Keywords:** breast cancer, trastuzumab, adjuvant, daily clinical practice

## Abstract

**Background:**

Human epidermal growth factor receptor 2 (HER2) positive breast cancer is an entity with aggressive behaviour. One year of adjuvant trastuzumab significantly improves the disease free survival in the range of 40–50% and reduces the risk of dying from HER2 positive breast cancer by one third. Adjuvant treatment with trastuzumab became available in Slovenia in 2005 and the aim of this study is to explore, if the exceptional results reported in adjuvant clinical trials are achieved also in daily clinical practice.

**Patients and methods.:**

An analysis of tumour and patient characteristics, type of treatment and outcome (relapse free and overall survival) of 313 patients (median age 52 years) treated at the Institute of Oncology Ljubljana in years 2005–2009 was performed.

**Results:**

Median follow-up was 4.4 years. Sixty-one patients relapsed and 24 died. Three and four years relapse free survival was 84.2% and 80.8% and the overall survival was 94.4% and 92.5%, respectively. Independent prognostic factors for relapse were tumour grade (HR 2.10; 95% CI 1.07–4.14; p = 0.031) and nodal stage (HR 1.35; 1.16–1.56; p < 0.0001) and for the overall survival nodal stage only (HR 1.36; 1.05–1.78; p = 0.021).

**Conclusions:**

The outcome in patients with adjuvant trastuzumab in daily clinical practice, treated by medical oncologists, is comparable to results obtained in international adjuvant studies.

## Introduction

The most common cancer in women in the developed world as well as in Slovenia is breast cancer (BC).^1^ With the introduction of the tumour gene signature the clinical observation that BC is a spectrum of different diseases in terms of prognosis and response to the treatment was confirmed.^2^ Using this tool as well as by classical clinico-pathological parameters four types of BCs can be distinguished and human epidermal growth factor receptor 2 (HER2) positive type is one of them, representing about 15% of newly diagnosed invasive BCs.^2^–^4^ It is a unique entity with an aggressive behaviour, characterized by over-expression of HER2 receptor and/or HER2 gene amplification.^3^,^5^ At the beginning of this century, trastuzumab, a humanized monoclonal antibody, that targets HER2 receptor, was approved for the treatment of patients with metastatic HER2-positive BC.^6^–^8^ Given the success of this antiHER2 drug in the metastatic setting, several large, randomized trials were initiated to evaluate its role in the early stage disease. The first results were presented in 2005 and were the basis for the approval of one year adjuvant treatment of patients with HER2 positive BC.^9^–^11^ A significant improvement in disease free survival (DFS) in the range of 40–50% was demonstrated and the risk from dying from BC was reduced by about one third. Reported 4-year DFS and the overall survival (OS) in the trastuzumab arms were 78.6–86% and 89.3–94%, respectively.^12^–^15^ This is the range of benefit seldom achieved in oncology. In the proceeding years new antiHER2 drugs confirmed their activity in metastatic setting; *i.e.* lapatinib, pertuzumab and trastuzumab-emtansine.^16^–^18^ Adjuvant studies with new antiHER2 drugs are in progress.

The adjuvant treatment with trastuzumab became available in Slovenia in 2005 and the aim of this report is to explore if these exceptional results reported in adjuvant clinical trials are achieved also in daily clinical practice.

## Patients and methods

With the approval of the adjuvant trastuzumab treatment the Slovenian HER2 registry was set up. The criteria for the adjuvant treatment with trastuzumab regarding tumour and nodal stage and cardiac function were the same as in pivotal adjuvant trials: tumours larger then 2 cm if node negative disease, any tumour size if node positive disease, performance status zero or one, no serious concomitant cardiac diseases and treatment with adjuvant chemotherapy.^9^–^11^ Data were collected from patient’s records. Patients were treated at the Institute of Oncology Ljubljana.

The study was approved by the institutional review board committed.

The main objective of this project was to evaluate the outcome of our real life patient population: relapse free survival (RFS) and OS. We compared our results with the results from randomized studies and other population-based studies.

### Statistical analysis

RFS was defined as time elapsed from date of surgery to date of the first relapse (local or distant), date of the last follow-up or date of death without relapse. Patients who died without relapse were censored at time of death. OS was defined as time from surgery to date of death of any cause or date of the last follow-up for patients who were alive. The univariate statistical analysis was performed using Kaplan-Meier method and log-rank test. The multivariate analysis was performed with Cox proportional hazards model. SPSS software version 16 was used for the statistical analysis.

## Results

In the 5-year period (2005–2009) 313 patients with HER2 positive BC were treated with adjuvant trastuzumab. The median age of the patients was 52 years (23–76). Median follow-up time was 4.4 years (minimum 0.2 years, maximum 6.9 years). The characteristics of the tumours are presented in Table 1. One hundred and twenty-seven (40%) of patients received an anthracycline-based and 165 (53%) anthracycline- and taxane-based chemotherapy. One hundred and seventy-six (56%) of patients had estrogen receptor (ER) and 130 (42%) of patients had progesterone receptor (PR) positive tumours. All patients with hormone dependent tumours (187 [60%]) were also treated with adjuvant endocrine therapy. Two hundred and seven (66%) patients were concomitantly with trastuzumab irradiated to the chest wall, breast and supraclavicular region, according to the international guidelines.^19^

### RFS - relapse free survival

**FIGURE 1.**
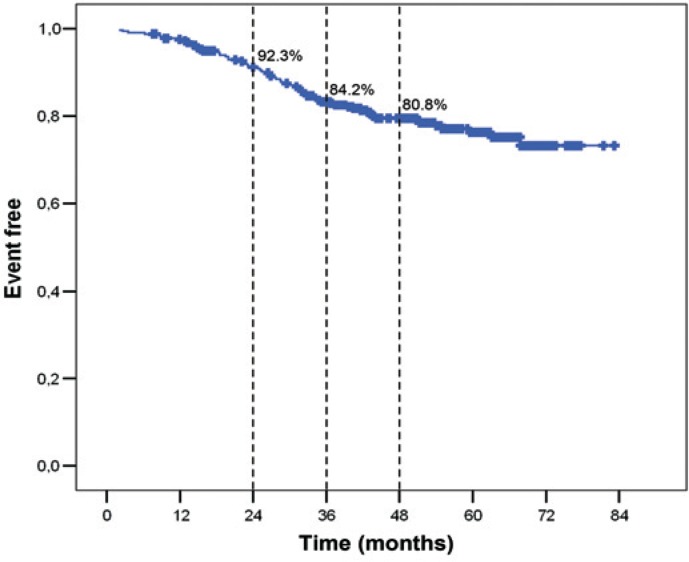
Relapse free survival (RFS).

Sixty-one patients (19.5%) relapsed. Kaplan-Mayer curve for RFS is presented on Figure 1. RFS at 4 years was 80.8%. Tumour stage and grade and nodal stage were found to have a significant impact on RFS in univariate analysis (Table 2). In the multivariate analysis only tumour grade (Hazard ratio [HR] 2.10) and nodal stage (HR 1.35) were found to have independent prognostic role (Table 3).

### OS – overall survival

**FIGURE 2.**
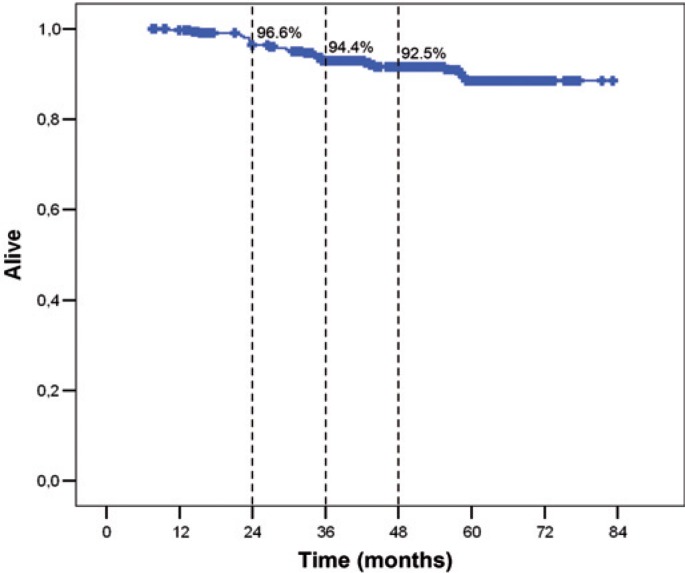
Overall survival (OS).

Twenty-four patients (7.6%) died. Kaplan-Meier survival curve is on Figure 2. OS at 4 years was 92.5%. For OS nodal stage was found to be the only statistically significant factor (HR 1.36) (Table 4).

## Discussion

The results of our institutional study are confirming the benefit of one year adjuvant trastuzumab treatment in daily practice. The magnitude of benefit was in the range of randomized studies; RFS at 4 years was 80.8% and OS 92.5%, respectively.

HER2 positive BC is a disease with an aggressive behaviour. Before the era of antiHER2 treatment the estimated 4–5 years OS rate was 75–87%. With the introduction of one year adjuvant trastuzumab the OS of these patients has improved significantly, according to the results of large international studies by about one third.^12^–^14^ In Slovenia, trastuzumab was rapidly implemented in the daily management after the release of these data, in the second half of 2005 already. It is well known that real life population is different to selected study population. In real world patients usually have more concomitant disease, are not as compliant as study population, cardiac follow-up is not done so often; all these factors can consequently result in worse results. The aim of our study was to assess the benefit of one year adjuvant trastuzumab treatment in our real life BC patients and to compare it with the results obtained in randomized clinical studies and other published population-based studies.

In Slovenia with two million inhabitants and about 1200 newly diagnosed BCs yearly at the time of the start of this retrospective observational study we had one comprehensive cancer centre, Institute of Oncology Ljubljana.^20^ This is important data since the adjuvant trastuzumab treatment was preceded at this institution only and not many patients were lost from registration and follow up in the database. The median age of 313 patients included in the study was 52 years. This is comparable to international studies in which 50 to 55% of patients were younger then 50 years.^9^–^14^ Our patients had larger tumours compared to patients in international studies if a B-31 part of North American study population is excluded in which node negative patients were not included.^11^ Seventy-six percent of patients had tumours T1 and T2 and only 25% of patients had node negative disease. In comparable international studies 80–90% of patients had tumours smaller then 5 cm. Thirty-three and 30% of patients had node negative disease in HERA and BCIRG 006 study, respectively.^9^,^10^ On the contrary, in the Dutch cohort of 479 HER2 positive BC patients 55% had node positive disease.^21^ The aggressiveness of this type of BC could be reflected by larger volume of the disease at the first presentation and higher tumour grade. There were 70% of high grade tumours in our population and this is comparable to North American study population.^11^ Only 14% of tumours were of low mitotic index. Regarding the hormonal receptor status our cohort of patients did not differ to historical cohorts.^9^–^11^ It is known that about 50% of HER2 positive BCs have positive estrogen and/or progesterone receptors and this was alike in our population.^22^

Adjuvant chemotherapy was as in clinical studies.^9^–^14^ Anthracycline-based chemotherapy was given before trastuzumab, taxane-based chemotherapy concurrently with trastuzumab. More than 90% of patients were treated with either anthracycline- or anthracycline- and taxane-based chemotherapy. Adjuvant endocrine therapy was prescribed according to international guidelines, after adjuvant chemotherapy and concomitantly with adjuvant trastuzumab. Locoregional radiotherapy was delivered to 66% of patients; the dose and the schedule were according to international guidelines.^19^

Tumour grade and nodal involvement were the only independent prognostic factors for the relapse (Table 3). Nodal stage was the only prognostic factors for OS. Also in North American study the tumour and nodal stage were found to be factors significantly important for both, DFS and OS. In that study especially patients with more than 10 lymph nodes involved were at the highest risk and gained the highest absolute improvement with adjuvant trastuzumab.^11^,^14^ Although RFS and DFS are not fully comparable (less events in RFS), the outcome of our non-study population is comparable to the results obtained in the international studies.^12^–^14^ The Dutch retrospective cohort study reports similar results; five years DFS and OS was 81% and 91%, respectively.^21^

Despite indisputable efficacy of adjuvant trastuzumab treatment some questions still remain. One of them, the optimal treatment duration, was mainly resolved after obtaining results of HERA study, which showed that two years of adjuvant treatment was not more effective than one year.^23^ Shorter regimens like in FinHER study^24^ were not confirmed in PHARE and ShortHER studies.^25^,^26^ St. Gallen consensus 2013 showed almost 100% agreement among panel discussant regarding one year lasting duration of the adjuvant trastuzumab treatment.^19^ There are new promising antiHER2 drugs, namely pertuzumab and trastuzumab-emtansine, which have already proven their effectiveness in the metastatic setting and will probably even improve the impressing results of trastuzumab.^17^,^18^

We think that our results also indicate the advantage of being treated by highly educated specialists (all treating physicians were medical oncologists), in high volume oncological center and with regular cardiac function evaluation. A prospective study of cardiotoxicity of trastuzumab in adjuvant setting is underway at our institution to show putative early and long term side effects.

## Conclusions

The prognosis of HER2 positive BC has improved significantly since the introduction of antiHER2 treatment. Our results based on the treatment of real-life BC patients with one year of adjuvant trastuzumab are comparable to the results obtained in international clinical studies.

## Figures and Tables

**TABLE 1.** Tumour characteristics of 313 patients

	**No.**		
Histology	IDC	297	95%
	ILC	6	2%
	Other	10	3%
Tumor grade	I	3	1%
	II	84	27%
	III	220	70%
	Unknown	6	2%
Mitotic index	1	44	14%
	2	94	30%
	3	131	42%
	Unknown	44	14%
Vascular invasion	Present	15	5%
	Absent	186	60%
	Unknown	112	35%
Hormonal receptor status	ER positive	176	56%
	ER negative	137	44%
	PR positive	130	42%
	PR negative	180	58%
	ER and PR negative	126	40%
	Unknown	1	0%
Tumour stage	T1	88	28%
	T2	152	48%
	T3	34	11%
	T4	8	3%
	T4d	25	8%
	Unknown	6	2%
Nodal stage	N0	79	25%
	N1	157	50%
	N2	51	16%
	N3	24	8%
	Unknown	2	1%

IDC = invasive ductal carcinoma; ILC = invasive lobular carcinoma

**TABLE 2.** Relapse free survival (univariate analysis)

	**HR (95% CI)**	**P value**
Tumour stage	1.25 (1,11-1.40)	< 0.0001
Nodal stage	1.42 (1,23-1.64)	< 0.0001
ER status	1.14 (0,68–1.90)	0.62
PR status	0.82 (0,49–1.36)	0.43
Tumour grade	1.91 (0,98–3.72)	0.059
Mitotic index	1.35 (0,89–2.03)	0.157
Histological type*	1.12 (0,99–1.27)	0.081
Vascular invasion	1.04 (0,98–1.10)	0.162
Chemo – type**	1.29 (0.85–1.98)	0.237

* invasive ductal carcinoma, invasive lobular carcinoma, other types

** anthracycline-based, anthracycline- and taxane-based, other types of chemotherapy

**TABLE 3.** Relapse free survival (multivariate analysis). No. of events: 61/313

	**HR (95% CI)**	**P VALUE**
Tumour grade	2.10 (1.07–4.14)	0.031
Nodal stage	1.35 (1.16–1.56)	< 0.0001
Tumour stage	1.19 (1.04–1.36)	0.014

**TABLE 4.** Overall survival (univariate analysis). No. of events: 24/313

	**HR (95% CI)**	**P VALUE**
Tumour stage	1.17 (0,94-1,46)	0.155
Nodal stage	1.36 (1,05-1,78)	0.021
ER status	1.22 (0,54-2,80)	0.633
PR status	0.87 (0,39-1,96)	0.733
Tumour grade	2.49 (0,75-8,26)	0.136
Mitotic index	1.63 (0,82-3,24)	0.162
Histological type*	1.12 (0,49-1,35)	0.211
Vascular invasion	1.01 (0,92–1,11)	0.821
Chemo – type**	0.84 (0,40–1,74)	0.632

* invasive ductal carcinoma, invasive lobular carcinoma, other types

** anthracycline-based, anthracycline- and taxane-based, other types of chemotherapy
